# Extra-Abdominal Applications of the Greater Omentum in Canine and Feline Surgery: Current Knowledge, Surgical Techniques, and Clinical Perspectives

**DOI:** 10.3390/ani16172654

**Published:** 2026-08-24

**Authors:** Dorian Zielonka, Magdalena Morawska, Yauheni Zhalniarovich

**Affiliations:** Department of Surgery and Radiology with Clinic, Faculty of Veterinary Medicine, University of Warmia and Mazury in Olsztyn, 10-719 Olsztyn, Poland; dorian.zielonka@uwm.edu.pl (D.Z.); eugeniusz.zolnierowicz@uwm.edu.pl (Y.Z.)

**Keywords:** greater omentum, omentalization, extra-abdominal transposition, pedicled omental flap, reconstructive surgery, thoracic surgery, wound healing, small animal surgery

## Abstract

Background: The greater omentum is a highly vascularized and biologically active structure with drainage, immunomodulatory, proangiogenic, and proregenerative properties. Although it is widely used in abdominal surgery, its extra-abdominal application in dogs and cats remains less standardized. Methods: This review summarizes the available veterinary literature on extra-abdominal omentalization in small animals, including case reports, case series, experimental studies, and selected comparative data, supplemented by relevant evidence from human reconstructive surgery. Results: Two main surgical strategies are described: transposition of a pedicled omental flap with preservation of vascular continuity and free omental grafting, with or without microvascular anastomosis. Reported applications include thoracic surgery, chronic and infected wounds, orthopedic conditions associated with impaired healing, head and neck reconstruction, and experimental neurosurgical procedures. The omentum may support healing by improving vascularization, filling dead space, promoting drainage, and contributing to infection control. Conclusions: Current evidence suggests that extra-abdominal omentalization may be valuable as an adjunctive technique in selected complex cases, particularly when infection, poor vascularization, dead space, or delayed healing are present. However, further clinical studies are needed to define indications, outcomes, and safety more precisely.

## Simple Summary

The greater omentum is a thin, fatty fold of tissue inside the abdomen that has a rich blood supply and can help control inflammation, drain fluid, and support healing. Because of these properties, surgeons have used it not only inside the abdomen but also in other body regions. This review explains how the greater omentum can be moved outside the abdominal cavity in dogs and cats and discusses where it may be useful, including the chest, difficult wounds, infected areas, bone healing problems, and selected procedures used to rebuild damaged tissues. The article also summarizes the main surgical techniques, possible benefits, limitations, and complications. Available veterinary evidence suggests that the greater omentum may be valuable in complex cases where standard treatment may fail, especially when infection, poor blood supply, empty spaces left after tissue removal, or delayed healing are present. However, many reports are based on single cases or small groups of animals. Further clinical studies are needed before these techniques can be recommended for routine use. A clearer understanding may help surgeons choose safer and more effective treatments for pets.

## 1. Introduction

The greater omentum (*omentum majus*) is an extensive, bilaminar fold of the peritoneum. It extends from the greater curvature of the stomach and the proximal portion of the duodenum. In dogs and cats, it covers the surface of the abdominal organs [1]. This structure is highly vascularized. The main blood supply to the greater omentum is provided by the gastroepiploic arteries, which form numerous connections with the visceral circulation [1,2]. Within the omentum, characteristic aggregates of lymphoid tissue, known as milky spots, are present and participate in the immune response within the peritoneal cavity [3,4,5,6,7,8]. The omentum also contains a dense network of lymphatic vessels, which accounts for its lymphatic drainage capacity [9].

In addition to its main immunomodulatory and drainage functions, the greater omentum also exhibits properties that support healing and regeneration [10]. Its rich vascularization improves the local blood supply to abdominal organs. It provides oxygen and substrates necessary for metabolic processes, while the omental tissue itself is a source of mediators that promote regeneration and tissue repair. Among these, the vascular endothelial growth factor (VEGF) is considered to play an important role [11]. In response to an inflammatory stimulus, so-called omental activation occurs, accompanied by increased expression of cytokines and factors that accelerate healing and neovascularization [10]. Stromal cells with mesenchymal characteristics, derived from adipose tissue, have also been detected within the greater omentum. These cells are capable of differentiation and secretion of paracrine factors, including the previously mentioned VEGF [12,13]. As a result, the omentum acts as an active biological material supporting healing. It migrates towards the site of injury, contributes to the control of infection and stabilization of the inflammatory focus, and at the same time improves local conditions for tissue repair [10].

Omentalization is used, among other indications, in the treatment of abscesses and cysts, for example within the prostate or in cases of uterine stump abscess, where drainage and improvement of the local healing environment are of particular importance [14,15,16]. The effectiveness of omentalization has also been described in the treatment of pancreatic abscesses as an alternative to maintaining open drainage of the pancreatic lesion [17]. In view of these observations, the beneficial effects of using the greater omentum in the treatment of intra-abdominal pathological lesions have provided the basis for attempts to apply it outside the abdominal cavity, both in veterinary medicine and in human medicine [18,19].

The description of the greater omentum as a “forgotten organ” appeared as early as the 1990s and drew attention to the surgical potential of this structure [20]. Contemporary publications have related these observations to small animal surgical practice [21,22]. In the context of extra-abdominal applications, particularly important properties of the greater omentum include its ability to support revascularization, modulate the immune response, and absorb fluids [10,11]. The scope of these possibilities is well illustrated by experience from human medicine, where the greater omentum has been routinely used in the treatment of extra-abdominal conditions [18,19]. Available review articles organize the current state of knowledge and indicate the uneven level of available evidence, ranging from experimental studies to case reports and small clinical case series [21,22,23].

**Figure 1:**
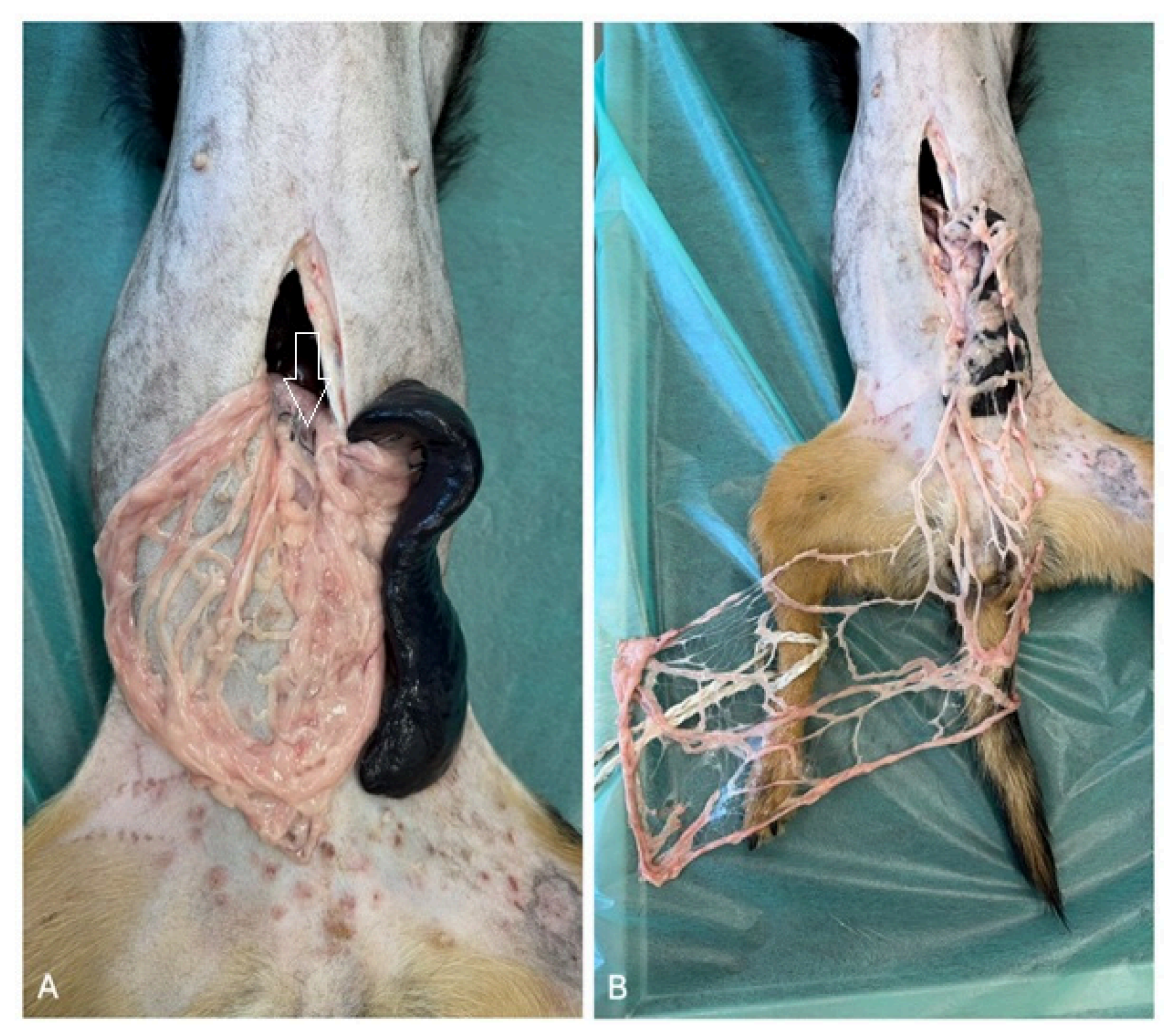
Cadaveric demonstration of the preparation and elongation of a pedicled greater omental flap in a dog. (**A**) The greater omentum immediately after exteriorization from the abdominal cavity, with the spleen visible adjacent to the omental tissue. The white arrow indicates the vascular pedicle that should be preserved during flap preparation. (**B**) The greater omentum after surgical elongation using an inverted L-shaped incision. The elongated flap demonstrates preservation of the vascular network while providing increased length for extra-abdominal transposition.

In reconstructive surgery, the greater omentum can be used outside the abdominal cavity in two main ways. The first involves transposition of a pedicled flap while preserving vascular continuity. The second involves the use of a free graft after excision of a fragment of the omentum and performance of microvascular anastomosis [19,24,25]. Classical omentoplasty consists of dissecting a portion of the omentum while preserving its vascular axis and transferring it to a target site outside the peritoneal cavity [20,22,24]. The gross appearance of the greater omentum after exteriorization from the abdominal cavity and after surgical elongation using an inverted L-shaped incision is shown in Figure 1 (Figure 1). Cadaveric studies in dogs have allowed more precise determination of the potential reach of the flap and the practical routes for its transfer [24]. Depending on the indication, the omentum may be passed through an opening in the diaphragm into the thoracic cavity or mediastinum [26], or the pedicled flap may be tunneled subcutaneously, for example through the substernal region [24,27].

If the reach of the pedicled flap proves insufficient, techniques for its elongation have been described. These include, among others, mobilization of the omentum in the region of the pancreas and an inverted L-shaped incision, which allows additional length to be obtained [24,27]. When the required distance exceeds the possibilities of a pedicled flap, microsurgical implantation of a free omental flap remains an alternative. Experimental studies have demonstrated the feasibility of transplanting a free fragment of the omentum and restoring its blood supply through microvascular anastomosis, for example to the medial saphenous artery and vein [25,28]. Successful transfer of a free flap to the distal portions of the limb in dogs has also been described [25]. However, this technique is rarely used in veterinary practice.

Subcutaneous tunneling is the most commonly described method of transferring a pedicled flap without the need for microanastomosis. The greater omentum can be passed beneath the skin from the open abdominal cavity to the selected body region [22,27,29]. Cadaveric studies indicate that the reach of an omental flap in dogs may be considerable. The mean length of the unmodified omentum was 25.8 cm, whereas after application of an elongation technique, the mean flap length reached 45.3 cm, with maximum values of up to 65 cm. This allows access to distant regions, such as the shoulder, axilla, and even the distal portions of the limbs [24]. In cases in which such reach remains insufficient, a free graft remains the available solution [25,28].

The use of the greater omentum, similarly to other reconstructive techniques, is associated with a risk of complications. In older veterinary reports, the most frequently emphasized complication was partial flap necrosis, usually related to impaired perfusion resulting from excessive tension, twisting, or compression of the vascular pedicle [30]. More recent data from human surgery confirm that the most important technical risks concern compromised flap vascularization and complications at the site of flap transfer or harvest. Liu et al. (2024) [31] described a group of 300 patients who underwent breast reconstruction using a laparoscopically harvested omental flap. The overall complication rate was 12.3%. The most commonly observed complication was subcutaneous fluid accumulation in the reconstructed breast region, including seromas or hematomas, which occurred in 7.0% of patients. Partial necrosis of the omental flap was reported in 3.3% of patients, and bleeding in 1.3%. Complications directly related to laparoscopic omental harvest were rare and included one case of vascular injury and one case of abdominal hernia, each corresponding to 0.3% of the study population [31]. The authors emphasized that flap necrosis was usually partial and most often resolved with conservative treatment, including prolonged drainage and local wound management. No cases of total flap necrosis, skin flap necrosis, nipple–areola complex necrosis, or wound infection were observed [31].

The authors attributed partial flap necrosis primarily to disturbances in flap perfusion. Anatomical variations of the gastroepiploic arcade may be relevant, as the peripheral portions of the omentum do not always receive sufficient blood supply from a single pedicle. The risk of ischemia may also increase during flap preparation and transfer, particularly when small omental vessels are damaged, when the flap is repeatedly pulled through a subcutaneous tunnel, or when excessive tension or compression occurs within the recipient bed. For this reason, the authors recommend careful assessment of vascularization in the peripheral portions of the omentum, gentle handling of the flap, and avoidance of situations in which the pedicle may be twisted, compressed, or placed under excessive tension [31]. Although laparoscopic harvesting may potentially reduce the morbidity associated with open celiotomy, veterinary experience remains limited. In dogs, laparoscopic-assisted partial omentectomy and omentopexy have been shown to be feasible with minimal perioperative morbidity. Laparoscopic mobilization and transposition of a pedicled omental flap have also been described in an individual clinical case. However, laparoscopic harvesting of an omental flap specifically for distant extra-abdominal reconstruction has not yet been standardized in dogs or cats [32,33].

Veterinary data are more limited and are derived mainly from small case series. However, the nature of the reported complications is similar. In cats with chronic axillary wounds treated with an omental flap, serous wound discharge, suppuration, and postoperative hernias have been described [34]. Brockman et al. also reported hernias associated with the site of flap exteriorization, including visceral herniation through a midline opening [35]. This indicates that not only the survival of the flap itself is important, but also the method by which it is transferred outside the abdominal cavity. In a more recent study involving 10 dogs treated with free omental grafts for extensive wounds of the distal limbs, a low number of complications and good clinical outcomes were reported. Nevertheless, infection, swelling or discharge, and partial graft loss occurred in individual cases within this group [35].

Complications related to the donor site and the site of flap exteriorization also deserve attention. In the limited veterinary literature, postoperative herniation was reported in 2 of 10 cats in one case series and in 2 of 5 cats in another. In the latter series, one cat developed intestinal herniation and one developed presumed abdominal fat herniation through the ventral abdominal exit site [34,35]. Because these findings are derived from only two small case series, the incidence of postoperative herniation following omental harvesting and transposition cannot be reliably estimated. A broader perspective is provided by data from human surgery. In a systematic review, Smit et al. (2024) [36] assessed the consequences of omentectomy and harvesting of the greater omentum for extra-peritoneal reconstruction. Among early complications, the authors primarily listed bowel obstruction, bowel stenosis, intra-abdominal abscesses, and sepsis, although their frequency varied among the analyzed studies and reached up to 23%. In long-term follow-up, the most important problem remained donor-site hernias, reported in up to 32% of patients, particularly after procedures performed through an open approach [36]. This means that complications of omentalization are not limited to the flap itself. They may also result from laparotomy, tissue harvest, and creation of a tunnel or passage to the recipient site [33,36]. However, it should be emphasized that in dogs and cats their true incidence remains difficult to determine, as the available veterinary data are based mainly on case reports and small clinical series.

Although the concept of transferring the greater omentum outside the abdominal cavity is attractive, in practice it is limited primarily by technical and anatomical factors. In most veterinary reports, the greater omentum has been harvested and shaped through an open celiotomy, making the procedure more extensive than many standard reconstructive techniques. Nevertheless, minimally invasive manipulation of the omentum has been demonstrated in dogs. Laparoscopic-assisted partial omentectomy and omentopexy were feasible in an experimental canine study, while laparoscopic-assisted mobilization of a pedicled omental segment through a chronic abdominal fistula was successfully performed in a clinical case [32,33]. These reports provide proof of technical feasibility but do not represent standardized laparoscopic harvesting of a pedicled or free omental flap for distant extra-abdominal reconstruction. Therefore, the extent to which laparoscopy could reduce donor-site morbidity in dogs and cats remains to be determined [32,33,36,37]. Individual patient-related factors are also important, including the amount of adipose tissue within the greater omentum. In cachectic or markedly underweight animals, the adipose component of the omentum may be limited, reducing its usefulness as a tissue for filling and stabilizing dead space. This is supported by necropsy observations in emaciated dogs, in which atrophy of omental fat has been described [38]. An additional limitation is the reach of the pedicled flap. In selected cases, it may be necessary to create a very long or complex subcutaneous tunnel, apply elongation techniques, or, in extreme situations, use a microsurgical approach [24,25]. Despite these limitations, reports have confirmed the feasibility of microsurgical free omental grafting in animals [25,28].

Experience from human surgery may help define potential directions for the development of extra-abdominal omentalization in small animals, as it includes larger patient populations and a broader range of reconstructive indications than the available veterinary literature. For example, omentalization is one of the methods used to treat infected sternal wounds after sternotomy in humans [39,40,41]. The omentum is also used to cover extensive chest wall defects and in selected reconstructions of the perineal region and other complex extra-abdominal defects [18]. These data indicate the potential of the omentum as a well-vascularized filling tissue that supports infection control, but they do not replace studies conducted directly in dogs and cats.

To organize the indications discussed further in this review, the main extra-abdominal applications of the greater omentum in dogs and cats, together with their potential mechanisms of action, level of available evidence, and key clinical limitations, are summarized in Table 1.

The aim of this article is to review the literature on extra-abdominal applications of the greater omentum in dogs and cats, taking into account experience derived from human medicine.

## 2. Materials and Methods

This narrative review was based on a structured search of the PubMed/MEDLINE, Web of Science, and ScienceDirect databases. The search covered publications available from database inception to July 2026 and was restricted to articles published in English. The search strategy used combinations of the following terms: “greater omentum”, “omentum”, “omentalization”, “omentalisation”, “omentoplasty”, “omental flap”, “omental graft”, “extra-abdominal”, “extraperitoneal”, “dog omentoplasty”, “canine omentalisation”, “cat omentoplasty”, “feline omentalisation”, “veterinary surgery”, “thoracic surgery”, “mediastinal omentalisation”, “wound omentalisation”, “reconstructive surgery”, “orthopedic surgery with omentalisation”, “bone healing”, “head and neck”, “neurosurgery”, “spinal cord”, “brain”, and “microsurgery”. Additional searches using the corresponding terms combined with “human” were performed to identify relevant evidence from human reconstructive surgery.

Titles and abstracts were screened for relevance, followed by assessment of the available full texts. Eligible publications included veterinary case reports, case series, clinical studies, experimental studies, cadaveric studies, and review articles describing extra-abdominal transposition, grafting, or microsurgical transfer of the greater omentum, as well as their indications, surgical techniques, outcomes, and complications. Studies involving dogs and cats were prioritized. Experimental studies in other species and human-medicine publications were included selectively when they provided relevant information regarding biological mechanisms, surgical techniques, flap perfusion, donor-site complications, or potential future applications not sufficiently addressed in the veterinary literature.

Duplicate publications, articles without sufficient relevance to surgical use of the greater omentum, and publications for which an English-language full text or sufficiently detailed abstract was unavailable were excluded. Studies concerning exclusively intra-abdominal applications were excluded from the main evidence synthesis unless they provided essential anatomical, physiological, or technical background. The reference lists of the included publications were also manually screened to identify additional relevant articles. Because of the heterogeneity of study designs and the predominance of case reports, small case series, and experimental studies, the findings were synthesized narratively, and no meta-analysis was performed. The strength-of-evidence categories presented in Table 1 were assigned descriptively according to the number and design of the available veterinary studies and should not be interpreted as a grading of treatment effectiveness or clinical outcome.

### 2.1. Use of the Greater Omentum in Thoracic Surgery

**Figure 2:**
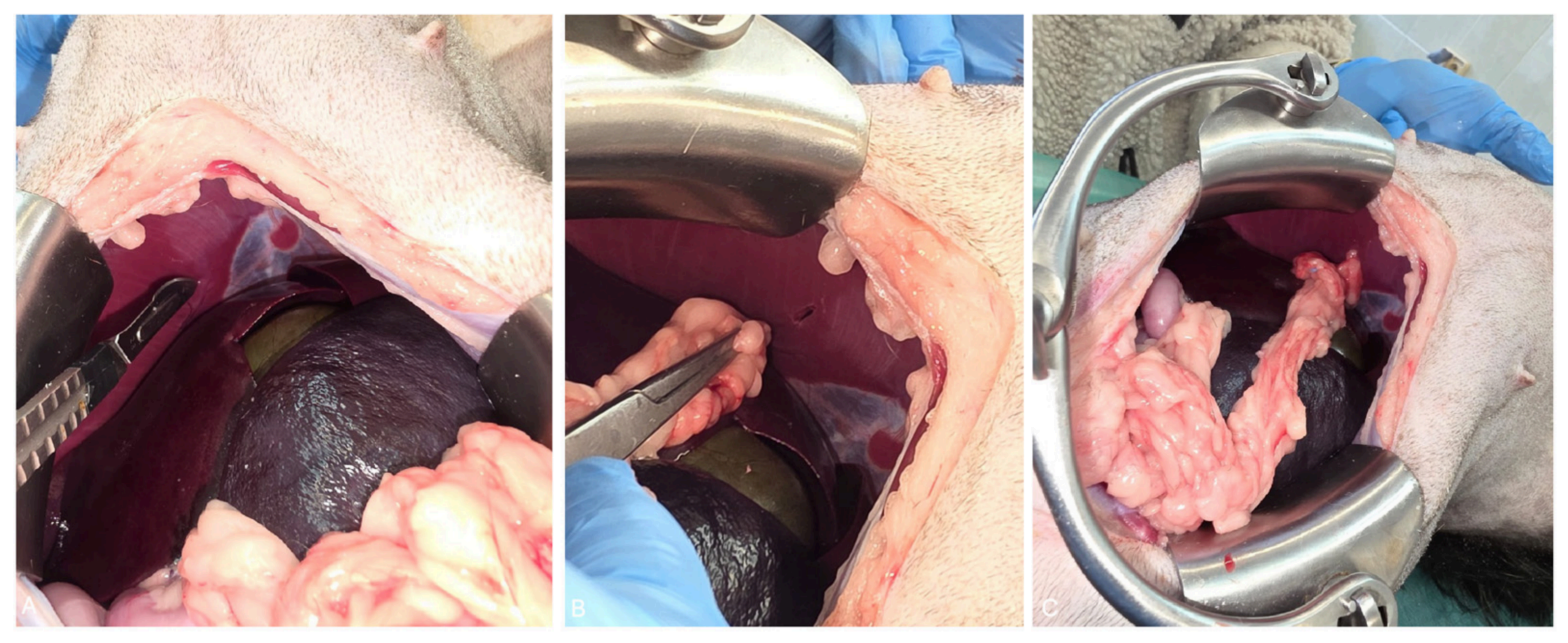
Transdiaphragmatic transfer of a pedicled greater omental flap into the thoracic cavity. (**A**) Intraoperative view of the thoracic cavity and diaphragm before transposition of the omentum; the intended site for creation of the diaphragmatic opening is shown. (**B**) Gentle traction of the greater omentum through the diaphragmatic opening. (**C**) The pedicled omental flap positioned within the thoracic cavity, demonstrating its potential use for thoracic omentalization.

**Figure 3:**
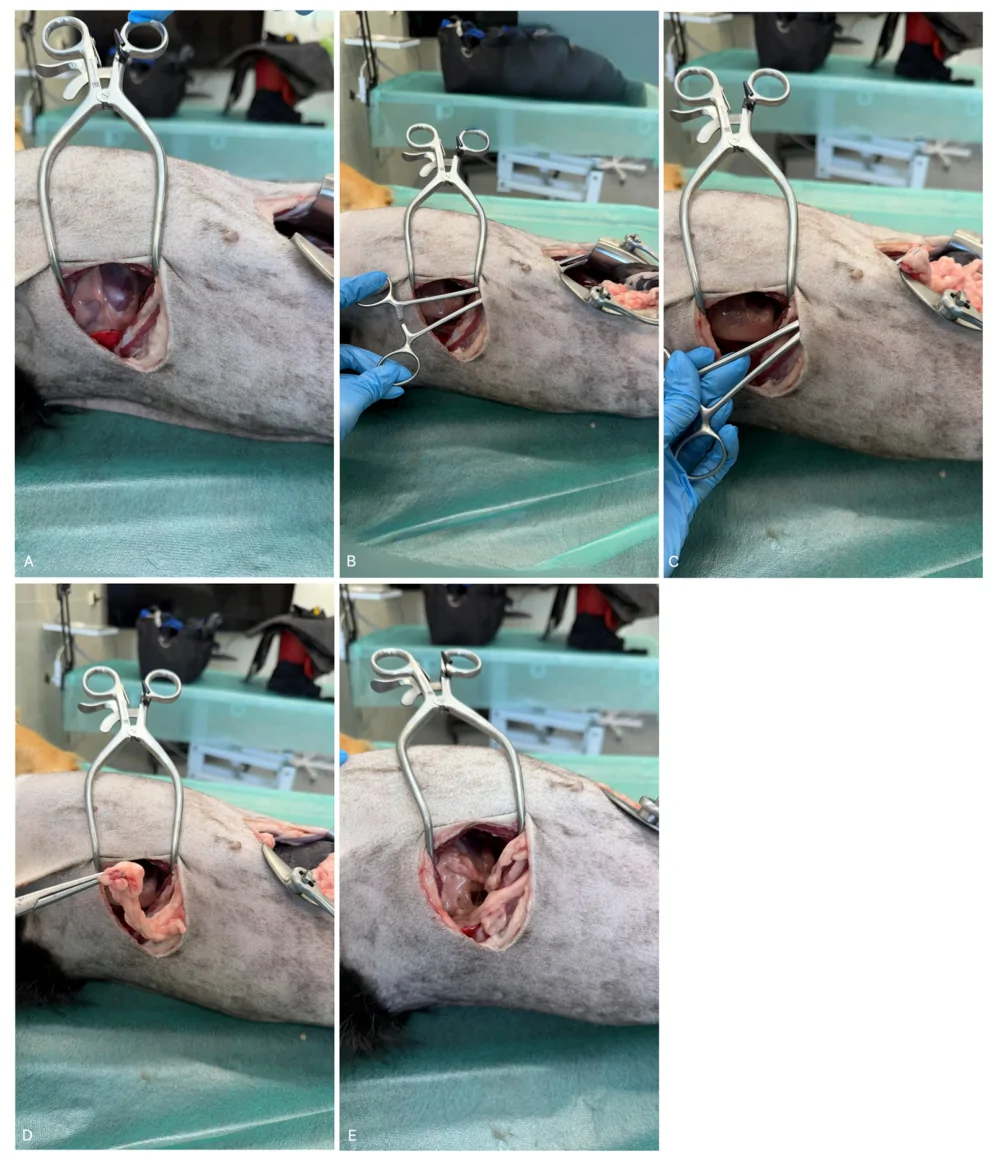
Subcutaneous transposition of a pedicled greater omental flap to the thoracic cavity through a lateral thoracic approach. (**A**) Intraoperative view after lateral thoracotomy, with the heart and lung lobe visible within the thoracic cavity; a ventral midline laparotomy is also visible. (**B**) Blunt creation of a subcutaneous tunnel between the laparotomy region and the lateral thoracic access site using a Pean forceps. (**C**,**D**) Retrieval of the greater omentum and gentle traction of the pedicled flap through the subcutaneous tunnel toward the lateral thoracic incision. (**E**) Final placement of the greater omentum within the thoracic cavity through the lateral thoracic approach.

**Figure 4:**
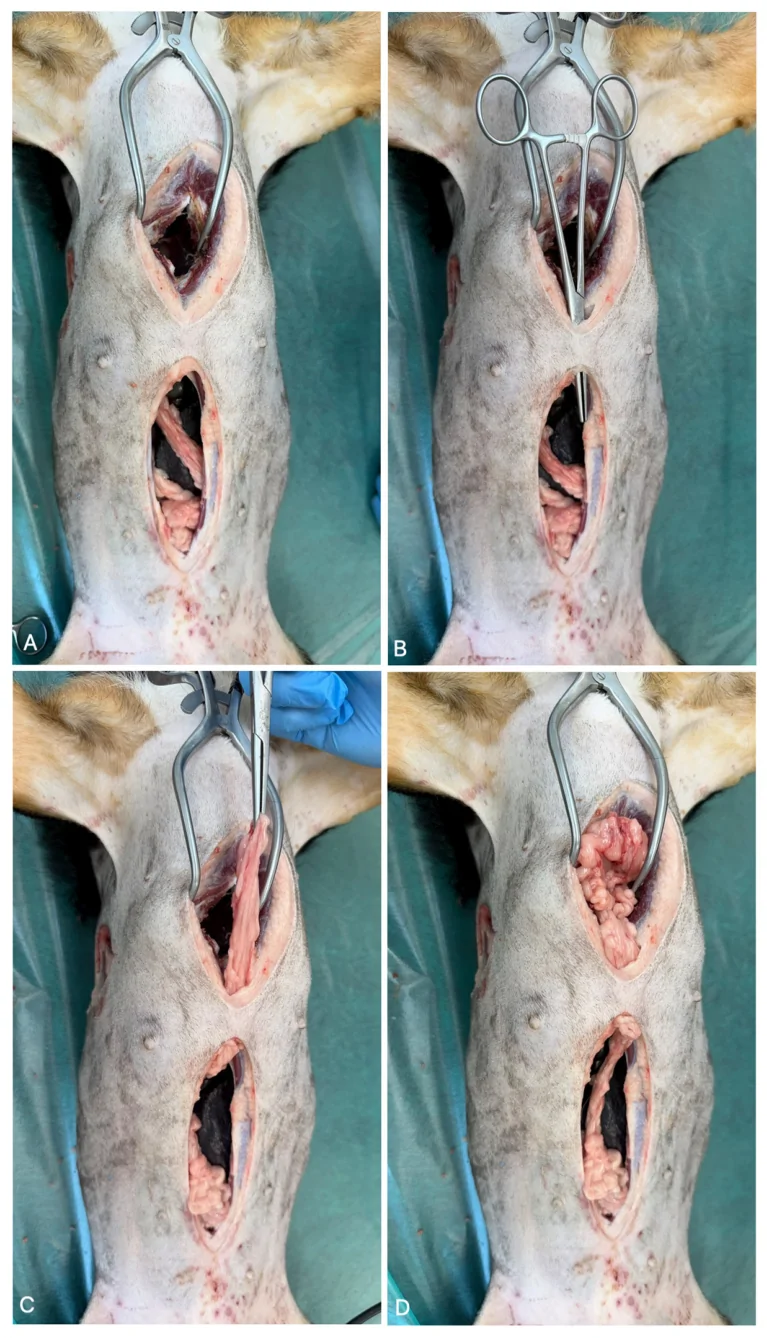
Subcutaneous transposition of a pedicled greater omental flap into the thoracic cavity through a sternal approach. (**A**) Intraoperative view showing the sternotomy and ventral midline laparotomy before omental transposition. (**B**) Blunt preparation of a subcutaneous tunnel between the laparotomy site and the sternal approach using Pean forceps. (**C**) Gentle traction of the greater omentum through the subcutaneous tunnel toward the thoracic access site. (**D**) The pedicled greater omental flap positioned within the thoracic cavity through the sternal approach.

Transposition of the greater omentum into the thoracic cavity may be performed using different routes, depending on the surgical indication, patient anatomy, and required flap reach. A pedicled greater omental flap can be advanced directly into the thoracic cavity through a diaphragmatic opening, thereby preserving its vascular pedicle and avoiding excessive tension or twisting (Figure 2). Alternatively, the omentum may be transferred through a subcutaneous tunnel created between the ventral midline laparotomy and a lateral thoracic access site (Figure 3) or introduced into the thoracic cavity through a median sternotomy combined with subcutaneous tunneling (Figure 4). These approaches illustrate the technical versatility of the greater omentum but also emphasize the need for careful flap handling and preservation of vascular supply during extra-abdominal transposition.

Idiopathic chylothorax in dogs and cats is a disorder characterized by the chronic accumulation of chyle within the pleural cavity due to leakage from the thoracic duct [74]. The most commonly described surgical treatment involves thoracic duct ligation (TDL), and in many protocols also partial pericardiectomy, which is intended to reduce venous pressure and improve lymphatic drainage [74,75]. Extended treatment approaches have also been described in the literature, including the combination of TDL with cisterna chyli ablation [76,77,78].

Omentalization of the thoracic cavity has been described as part of a strategy aimed at improving the removal of chyle from the pleural cavity by utilizing the absorptive properties of well-vascularized omental tissue [42,43,44]. In the report by Williams and Niles, the omentum was used in a dog as a pedicled flap and was passed into the pleural cavity through an opening in the diaphragm, thereby providing a form of physiological drainage [43]. Stewart and Padgett described the treatment of chylothorax based on TDL combined with omentalization, indicating that this combination may simultaneously reduce chyle inflow and facilitate its resorption from the pleural cavity [42]. In the study by Bussadori et al., the procedure was performed as a combined protocol including thoracic duct ligation, partial pericardiectomy, and thoracic omentalization [44]. The authors reported that, during the follow-up period, no recurrence of clinical signs was observed in 7 of 9 dogs and in 3 of 4 cats. At the same time, the conclusions of the study indicated that the inclusion of omentalization in the protocol did not provide a clear advantage over outcomes reported for standard treatment protocols for chylothorax [44]. Similarly, in a review article, Reeves et al. emphasized that the durability of the outcome in idiopathic chylothorax depends primarily on the appropriate selection and combination of surgical techniques, such as TDL, pericardiectomy, and other procedures, whereas the role of omentalization was considered supportive [74].

In thoracic infections, including pyothorax, effective evacuation of purulent exudate from the pleural cavity, control of the source of infection, maintenance of adequate drainage, and appropriately selected antimicrobial therapy are of key importance [45,79,80]. In their review, Stillion and Letendre emphasized that treatment may be either conservative or surgical, and that the choice of method depends on the patient’s condition, the presence of chronic lesions, and the response to initial management [79]. Clinical data indicate that surgery is usually recommended when infection persists despite drainage, antimicrobial therapy, and pleural lavage, or when structural abnormalities are present, such as adhesions, fibrinous fluid-filled loculations, foreign bodies, or proliferative pleuritis [45,46,79].

A more directly documented indication for thoracic omentalization is the treatment of caudal mediastinal paraesophageal abscesses. Franklin et al. described a 2-year-old, 25-kg German Shorthaired Pointer presented with acute lethargy, pyrexia, and tachypnea. Thoracic radiography and ultrasonography revealed a large, approximately 150-mm, multiloculated fluid-filled mass in the caudal mediastinum. Fine-needle aspiration yielded purulosanguineous material containing neutrophils and bacterial rods, whereas esophagoscopy did not reveal esophageal perforation or a foreign body. Surgical treatment was performed through median sternotomy. Because complete excision was not possible owing to close adhesion of the abscess to the diaphragm and mediastinal structures, the cavity was evacuated, lavaged, and partially debrided. A pedicled greater omental flap was then advanced through a 15-mm diaphragmatic opening and positioned within both chambers of the abscess cavity to provide vascularized tissue and physiological drainage. The dog recovered without major complications, and no respiratory impairment or exercise intolerance was reported at the 3-month follow-up [45].

Brissot et al. subsequently reported a case series of seven dogs with caudal mediastinal paraesophageal abscesses. All dogs presented with pyrexia and lethargy, six had regurgitation, and five had coughing. Thoracic computed tomography, performed in six dogs, consistently demonstrated a large fluid-filled structure extending from the caudal aspect of the heart toward the diaphragm and closely associated with the esophageal wall. Surgical access was obtained through median sternotomy in five dogs and lateral thoracotomy in two. In all cases, the abscess was opened, drained, lavaged, and partially debrided; complete excision was not possible because of its close relationship with the esophagus and aorta. In five dogs, the residual cavity was packed with greater omentum advanced through a diaphragmatic incision. All seven dogs survived surgery and were discharged after 4–11 days. No recurrence of the original clinical signs was reported during follow-up, and all dogs were alive six months after surgery. However, the two dogs treated without omentalization had outcomes similar to those of the omentalized dogs. Therefore, although the procedure appeared safe and may have supported drainage and healing, its specific contribution could not be distinguished from the effects of surgical drainage, debridement, thoracic drainage, and antimicrobial therapy [46].

These observations are further supported by more recent microbiological data, which emphasize the importance of pleural fluid culture and targeted antimicrobial therapy. Heinsoo et al. (2023) [80] analyzed 53 dogs with pyothorax, comparing culture and antimicrobial susceptibility results with recommended empirical treatment protocols. Pleural fluid culture was performed in 52 dogs, and a positive result was obtained in 30 of them, corresponding to 57.7% of patients. The most commonly isolated organisms were Pasteurella spp. and Escherichia coli, each accounting for 23.3% of positive cultures, as well as mixed anaerobic flora, which was identified in 20% of cases. The authors demonstrated that the bacterial composition of pyothorax is diverse and that resistance to some empirically used antimicrobials may be clinically relevant. This was particularly true for clindamycin, to which 69% of the evaluated aerobic isolates were resistant, including all tested Gram-negative bacteria. These findings indicate that clindamycin should not be used as the sole antimicrobial agent in the treatment of canine pyothorax, and that antimicrobial selection should be verified on the basis of culture and susceptibility testing [80].

Against this background, thoracic omentalization appears to be a technique reserved for selected cases, especially chronic, recurrent, or complicated ones. It may be useful after decortication or surgical debridement, when dead space remains despite removal of pathological tissue, when advanced fibrosis persists, or when improvement of the local healing environment is required [47]. However, the available veterinary data do not support considering it a standard treatment for pyothorax. It should rather be regarded as an adjunctive technique used in the most challenging cases, rather than as a routine component of management [45,46,47,79,80].

Less typical applications of the greater omentum within the thoracic cavity have also been described. In a cat with chronic neoplastic pleural effusion, thoracotomy and omentalization were used as a palliative procedure when regular evacuation of the pleural cavity was required and other methods failed to provide sustained improvement. After thoracotomy, the omentum was transferred into the pleural cavity to increase fluid resorption and reduce the need for repeated thoracocentesis. Following surgery, a reduction in the volume of effusion and an improvement in quality of life were achieved; however, the method had no effect on the course of the primary disease [48]. From a practical perspective, omentalization should therefore be regarded as a palliative and adjunctive procedure. At present, intermittent or permanent drainage, including the use of pleural access systems, remains a more commonly used approach for chronic pleural effusions [81,82].

For chronic or recurrent pleural effusions, fully implantable pleural access ports represent a less invasive alternative to thoracic omentalization because they allow repeated, controlled drainage without celiotomy and transposition of omental tissue [81,82]. However, pleural ports require repeated external access and do not provide vascularized tissue within the thoracic cavity. In contrast, omentalization may promote continuous fluid absorption and improve local healing conditions, but it requires a more invasive surgical procedure and does not affect the progression of the underlying disease [48,81,82]. Therefore, pleural access ports may be preferable when repeated palliative drainage is the primary goal, whereas omentalization may be considered selectively when physiological drainage and vascularized tissue support are desired.

In experimental studies, the greater omentum has also been used to support healing of esophageal anastomoses. Hayari et al. (2004) [49] evaluated the effect of omentopexy on the healing of esophagoesophageal anastomosis in a canine model. In 6 dogs, a right-sided thoracoabdominal incision was performed, a 5-cm segment of the middle portion of the esophagus was resected, and continuity was then restored by end-to-end anastomosis. In half of the animals, the anastomotic site was additionally wrapped with a pedicled flap of the greater omentum, which was passed into the thoracic cavity through an enlarged opening in the region of the right crus of the diaphragm [49]. The authors did not observe anastomotic leakage in either group, suggesting that omentopexy did not primarily affect the integrity of early healing. The differences concerned, instead, the quality of healing and subsequent esophageal patency. In the omentopexy group, 2 of 3 dogs returned to eating solid food, whereas none of the dogs in the control group tolerated solid food. Fluoroscopic examination showed preserved esophageal motility and faster passage of ingesta through the anastomotic site in animals treated with omentopexy, whereas dogs without omentopexy showed anastomotic stenosis, prestenotic esophageal dilation, and delayed emptying. Histological examination demonstrated neovascularization within the anastomotic region in the omentopexy group, whereas marked fibrosis predominated in the control group [49]. These findings indicate that the greater omentum may not so much prevent leakage as improve local vascularization and the quality of healing, thereby reducing the risk of subsequent esophagoesophageal anastomotic stricture.

A similar rationale has been applied in studies on bronchial and tracheal surgery. In a lung transplantation model, the omentum was used to cover the bronchial anastomosis and support its revascularization. Post-mortem examinations confirmed restoration of bronchial vascularization by the omental flap, and anastomotic stenosis occurred in only one dog [50]. In studies on the trachea, it was shown that placing the tracheal anastomosis in the well-vascularized environment of the greater omentum improved its survival and helped maintain patency [51]. These findings suggest that the omentum may reduce ischemia, wall necrosis, and subsequent stenosis, which are the main complications of tracheobronchial anastomoses [50,51]. However, it should be emphasized that these are mainly experimental reports. They demonstrate the considerable biological potential of the greater omentum, but do not yet constitute an indication for routine use of these techniques in small animal clinical practice.

### 2.2. Use of the Greater Omentum in Orthopedics

One of the most promising extra-abdominal applications of the greater omentum in veterinary medicine is the support of bone union and the treatment or prevention of infection in orthopedic surgery. Transfer of the omentum to a fracture site or to a limb wound requires bridging the distance between the abdominal cavity and the target region. This is most commonly achieved by subcutaneous tunneling. Excessive tension and twisting of the omental pedicle must be avoided [24]. If an increased reach of the omental flap is required, elongation techniques may be used, such as an inverted L-shaped incision [24,27]. If the reach remains insufficient despite these measures, microsurgical transplantation of a free omental fragment and vascular anastomosis to vessels near the recipient site may be performed [25,28].

In toy-breed dogs, fractures of the distal radius and ulna are associated with a high risk of delayed union or non-union. This is partly related to the specific vascular anatomy of these bones [83]. The use of well-vascularized omental tissue at the fracture site may theoretically improve prognosis by increasing the delivery of blood, cells, and reparative mediators to the injured area [52,84]. Angiogenesis is one of the fundamental prerequisites for normal fracture healing, and its inhibition leads to impaired bone union [85]. VEGF also plays an important role in this process [53,86,87,88,89].

The importance of improving local vascularization was also demonstrated in an experimental study by Bader (2011) [53], which evaluated the effect of a pedicled greater omental flap on the healing of non-fixed rib fractures in dogs. In the group in which the fracture site was covered with omentum, the fracture line disappeared earlier, callus remodeling began sooner, and the callus was smaller and more compact than in the control group. The author suggested that this effect may result both from improved vascularization and enhanced osteogenesis, as well as from a partial stabilizing effect of the omental flap [53].

In one of the few prospective, randomized, controlled clinical studies, Ree et al. (2018) [52] compared a free autogenous greater omental graft with a conventional autogenous cancellous bone graft in dogs weighing less than 6 kg with traumatic radial and ulnar fractures. This was a patient population particularly predisposed to delayed union, non-union, and refracture, which the authors associated, among other factors, with the small amount of soft tissue surrounding the bone and the limited density of intraosseous vessels in the radius and ulna of small dogs. The authors assessed the rate of radiographic union, complications, bone density, and vascularization of the fracture region using Doppler ultrasonography, computed tomography, and radiography. In the group treated with the greater omentum, radiographic union occurred faster than after cancellous bone grafting, with median healing times of 9 and 12 weeks, respectively, and the dogs began to bear weight on the operated limb earlier. No major complications related to harvest or use of the omental graft were reported, although local swelling, erythema, and serous discharge in the region of the operated limb were observed more frequently. Doppler ultrasonography showed that, in the omental graft group, a stronger vascular signal and a greater number of vessels within the developing reparative tissue persisted for longer, suggesting that the beneficial clinical effect resulted primarily from improved local vascularization [52].

Similar conclusions can be drawn from experimental studies. Saifzadeh et al. (2009) demonstrated, in a canine radial non-union model, that a free, non-vascularized greater omental graft placed in the region of the osteotomy gap promoted bone bridging and led to union, whereas features of non-union persisted in the control group [54]. Subsequent studies in dogs also evaluated more complex tissue-engineered constructs. In a radial bone defect model, Bigham-Sadegh et al. (2012) [55] compared an empty defect, omentum alone, omentum combined with culture medium, and omentum combined with adipose-derived stem/stromal cells, referred to as ASCs or ADSCs. All animals underwent radial stabilization using a bone plate and screws. The best radiographic and histopathological results were obtained in the groups in which the omentum was combined with additional biological factors, particularly adipose-derived cells or culture medium [55].

In another study, Bigham-Sadegh et al. (2013) [56] used a free periosteal graft wrapped in a pedicled greater omentum within the abdominal cavity of a dog. The authors showed that combining periosteum with omentum resulted in more pronounced formation of mature bone tissue than placement of the periosteal graft subcutaneously [56]. Subsequently, Bigham-Sadegh et al. (2013) [56] evaluated a construct combining pedicled omentum, periosteum, and autogenous or allogeneic adipose-derived stem cells. Bone formation was achieved in all groups, and the authors emphasized the role of the omentum as a well-vascularized scaffold that supplies oxygen, nutrients, and angiogenic factors, thereby creating a favorable microenvironment for graft survival and osteogenic cell differentiation [57].

Although these studies are experimental, they clearly demonstrate that the greater omentum may act as a biological scaffold supporting angiogenesis, survival of transplanted tissues, and bone formation. In relation to clinical small animal surgery, these findings should be interpreted with caution; nevertheless, they provide an important rationale for further evaluation of the omentum as an adjunctive technique to support healing of fractures at risk of delayed union or non-union [54,55,56,57].

Similar observations have also been reported in canine arthrodesis. Ree et al. (2016) [58] evaluated the use of a free autogenous greater omental graft as an adjunct to conventional arthrodesis with cancellous bone grafting. The analysis included cases of pancarpal arthrodesis, pantarsal arthrodesis, and partial tarsal arthrodesis. In the group treated with an additional omental graft, radiographic union scores were better at 9–12 weeks, whereas major postoperative complications requiring revision surgery occurred more frequently in the group treated without omental grafting [58].

In clinical practice, omentalization has also been described as an adjunctive treatment for infected femoral non-union in a dog. In this case, treatment was performed in two stages. First, the infected focus was surgically debrided. Omentalization was then used as part of the treatment of a biologically inactive non-union. In the subsequent stage, the fracture site was stabilized with a plate and an autogenous cancellous bone graft was applied. Bone union was confirmed radiographically after 16 weeks, and after one year the dog returned to full physical activity without lameness. This case report clearly illustrates that, in orthopedic conditions complicated by infection, mechanical stabilization alone is usually insufficient. Simultaneous measures aimed at infection control, improvement of vascularization, and restoration of biological activity at the non-union site are necessary [59].

The available data therefore suggest that the greater omentum may be particularly useful in three situations: first, in fractures and non-unions at risk of ischemia; second, in infected surgical fields, where infection control must be achieved in addition to bone union; and third, in the reconstruction of bone defects, when a well-vascularized environment is required for healing or for more advanced tissue-engineering approaches [52,54,55,56,57,58,59,60]. However, it should be emphasized that the number of clinical case reports in dogs and cats remains small. Most data are derived from experimental models. Therefore, omentalization in orthopedics should not currently be regarded as a routine method, but rather as an adjunctive technique for selected, challenging cases.

### 2.3. Use of the Greater Omentum in Reconstructive Medicine

The greater omentum is used in the treatment of difficult-to-heal chronic wounds and extensive soft tissue defects, particularly when local vascularization is impaired. The problem of chronic, non-healing wounds is especially evident in cats, in which the healing process is slower and differs from that in dogs [90]. In such cases, omentalization may accelerate wound cleansing and stimulate granulation and epithelialization [68,91,92]. Brockman et al. (1996) [35] described five cases of cats with chronic wounds in which, after wound debridement, the greater omentum was used as part of the reconstructive procedure. In all cases, the wounds healed completely, and the mean follow-up period was 2.5 years. However, the authors also reported complications, including hernias at the site of flap exteriorization and seroma formation, indicating that the effectiveness of this method is associated with the risk of additional invasiveness [35]. In a larger case series involving 10 cats, Lascelles et al. (1998) [34] used a pedicled omental flap to treat non-healing axillary wounds. Long-term healing was achieved in 7 of 10 animals, and final wound healing occurred at a mean of 24 days after omentalization. At the same time, wound dehiscence recurred in 8 cats, requiring at least one additional attempt at wound closure. Postoperative hernias, serous wound discharge, and an abscess in the paracostal region were also reported [34]. These findings suggest that the omental flap itself may be effective, but in more advanced cases it is not always sufficient as the sole reconstructive method.

This is supported by the study by Lascelles and White (2001), who combined a pedicled omental flap with a thoracodorsal muscle flap to close a chronic axillary wound in a cat [67]. In this study, all wounds ultimately healed, and the mean follow-up period was 21.7 months, although two cats required additional surgical intervention because of partial flap dehiscence. Such an approach appears particularly justified when the omentum improves the biological conditions for healing, but mechanical and durable coverage of a large skin defect is also required. A similar rationale was applied in the case report by Gray (2005), in which, after excision of chronic granulation tissue, an omental flap was tunneled subcutaneously to the axillary region and then combined with an omocervical flap, resulting in final healing of the chronic wound [69]. In practice, this means that the best outcomes were usually achieved not when the omentum was used instead of reconstructive techniques, but when it served as biological support for more conventional methods of wound closure [67,69]. Thus, the relatively greater volume of published veterinary evidence in this area should not be interpreted as evidence of consistently favorable clinical outcomes, as wound recurrence, dehiscence, herniation, discharge, and the need for additional surgical procedures were reported in several cases.

In dogs, the use of the omentum for wound treatment has been reported less frequently than in cats. This may partly be because chronic wounds with a persistent dead-space pocket, also referred to as an indolent pocket, are a well-recognized problem in cats. In these wounds, tissues adhere poorly to one another, and despite repeated attempts at closure, wound edges readily dehisce [91,93]. In dogs, the greater omentum has been used mainly when, in addition to wound closure itself, improvement of local vascularization, filling of dead space, and support of infection control were required. Large open wounds of the distal limbs are a good example. Makar and Baltzer (2025) described 10 cases in dogs in which, after harvesting the omentum by laparotomy, a free graft was sutured directly to the wound bed and to the subcutaneous tissue beneath the skin edges [70]. The authors obtained good functional and cosmetic outcomes with a low number of complications, suggesting that a free omental graft may be useful in the reconstruction of challenging distal limb wounds, where the options for conventional pedicled flaps are limited.

The greater omentum has also been used in dogs with chronic sublumbar abscesses and draining tracts. In a case series, Woodbridge et al. (2014) [71] performed omentalization of the abscess cavity after surgical debridement of the lesion. The aim was not only to remove inflammatory material, but also to fill the remaining cavity with well-vascularized tissue that improves drainage, increases local blood supply, and limits the persistence of dead space. During long-term follow-up, clinical signs resolved in all dogs, and the number of complications was low [71]. A similar rationale was used by Birettoni et al. (2017) [72] in cases of sublumbar abscesses associated with migrating plant material. The authors performed a ventral midline laparotomy, removed the foreign body under intraoperative ultrasonographic guidance, and additionally omentalized larger abscess cavities [72]. In these cases, drain placement was not required, and all dogs regained full function within 4–5 weeks. Both studies therefore suggest that, in deep sublumbar abscesses, omentalization may be particularly useful when, after debridement, a large, infected cavity remains that is difficult to manage effectively with drainage alone [71,72].

In more complex cases, the role of the greater omentum may extend beyond simply filling an infected cavity. Thatcher et al. (2024) described a dog with chronic discospondylitis, osteomyelitis, a sublumbar abscess, and a pathological vertebral fracture, in which ventral omentalization was combined with simultaneous dorsal stabilization [73]. This case differed from earlier reports concerning simpler sublumbar abscesses because, in addition to infection control, mechanical instability also had to be addressed [71,72,73]. In this context, the greater omentum served as biological support for healing, whereas stabilization provided the mechanical conditions necessary for successful treatment [73]. This report suggests that, in the most severe cases, omentalization should not be regarded as a stand-alone method, but rather as one component of combined treatment integrating infection control, elimination of dead space, and restoration of tissue stability.

### 2.4. Use of the Greater Omentum in Head and Neck Conditions

The use of the greater omentum in head and neck surgery in small animals represents a major challenge because of the considerable anatomical distance between the abdominal cavity and the target region [19,22]. Cadaveric studies indicate that the omental flap may reach the cervical region, and, when specific elongation techniques are used, even the head region in cats; however, this is associated with a substantial risk of flap injury or ischemia [61]. In veterinary clinical practice, there are no reports of routine use of the greater omentum in head and neck reconstruction. Attempts described to date have been mainly experimental or anatomical in nature [21,22]. Nevertheless, isolated reports from veterinary reconstructive microsurgery and esophageal reconstruction indirectly support the feasibility of such procedures. Degner et al. (1996) demonstrated the possibility of transferring free myoperitoneal flaps in dogs, while Lee et al. (2008) described a case in which a diaphragmatic flap combined with an omental flap was used for esophageal reconstruction in a dog, with the omentum serving as a well-vascularized tissue that covered and supported healing at the reconstruction site [62,63]. These data suggest that the main limitation of using the greater omentum in the head and neck region in small animals is not so much the lack of biological potential of this tissue, but rather the substantial technical difficulty and the limited number of clinical experiences.

In human medicine, by contrast, the greater omentum has been used for many years in complex reconstructions of this region. Both free omental flaps and gastro-omental flaps have been described for reconstruction of extensive defects of the oral cavity, pharynx, and neck [94,95,96]. In individual cases, reconstruction of the floor of the mouth has been performed using a gastro-omental flap [97], and other oral cavity defects have been reconstructed using a free flap containing the greater omentum [98]. Simultaneous reconstruction of complex neck defects together with a segment of the esophagus using a composite gastro-omental flap is also possible [99,100]. In these indications, however, gastro-omental flaps are not unequivocally superior to all other reconstructive techniques. In reconstructions of the oral cavity and oropharynx, they have mainly been compared with the radial forearm free flap; no significant differences in overall clinical outcome were demonstrated between these methods, although long-term swallowing disorders remained a common problem regardless of the technique used [101]. This means that, in typical mucosal reconstructions, the gastro-omental flap has not replaced standard methods, such as the radial forearm flap, which remains the primary option in many centers.

The particular value of the greater omentum becomes apparent in more complex cases, especially in a so-called hostile wound environment, that is, in tissues previously subjected to radiotherapy or multiple surgical procedures, in the presence of fistulas, infection, or extensive dead space, or when simultaneous mucosal reconstruction and soft tissue coverage of the neck are required [102,103,104]. In such situations, the advantages of the gastro-omental flap include excellent vascularization, substantial tissue pliability, and the ability to fill irregular defects. In patients with unfavorable hypopharyngeal defects and in analyses of circumferential pharyngeal reconstructions, this method has been shown to provide good functional outcomes, but it is also associated with its own complication profile, including the risk of fistula formation and stenosis [102,103]. In practice, this means that the gastro-omental flap is not a first-choice technique for every head and neck defect, but rather a specialized reconstructive option for particularly challenging cases.

Similar conclusions can be drawn from analyses of cervical esophageal fistula repair. In such situations, the pectoralis major muscle flap is commonly used as a salvage option; however, Reid et al. (2004) demonstrated that a free omental flap may provide a better reconstructive effect in particularly difficult cases complicated by previous anterior cervical spine fixation [105]. This suggests that the advantage of the omentum may become most evident when thin, highly vascularized tissue is needed to simultaneously isolate an infected field, fill dead space, and improve local healing conditions.

Against this background, the situation in veterinary medicine is different. In maxillofacial reconstruction and after oncological resections in dogs and cats, local and regional flaps currently predominate, as they are technically simpler, do not require laparotomy or microanastomosis, and in most cases allow satisfactory coverage of the defect [106]. Omentalization in the head and neck region in small animals therefore remains a technically feasible but experimental method. It may be assumed that, with the development of microsurgery and more advanced reconstructive techniques, its use may increase; however, at present, there are no clinical data that would allow it to be considered a routine procedure.

### 2.5. Use of the Greater Omentum in Neurosurgery

One of the most interesting, although still largely experimental, applications of the greater omentum is the support of nervous system regeneration [107,108,109]. This concept is based on the assumption that well-vascularized and biologically active omental tissue may increase perfusion of ischemic or damaged neural structures, limit the extent of secondary injury, and create an environment favorable for tissue repair [109]. Goldsmith and colleagues developed this idea in relation to both the spinal cord and the brain, considering omentalization as a method of revascularizing damaged areas of the central nervous system [109,110,111].

With regard to the spinal cord, both experimental studies and clinical attempts in humans have been described. Experimental models have shown that early transfer of a pedicled omental flap to the site of spinal cord injury may increase blood supply to the damaged tissue and limit the extent of secondary post-traumatic injury [64,112]. In clinical studies involving patients with chronic spinal cord injury, laparotomy was performed, the omental flap was elongated, and, after laminectomy, it was passed into the vertebral canal and placed directly on the exposed spinal cord [110,113]. Older reports described some neurological improvement in selected patients; however, a later prospective study by Duffill et al. (2001) did not demonstrate significant improvement in neurological function in 17 patients with chronic spinal cord injury, although postoperative imaging confirmed survival and persistence of the omental flap [113]. In addition, complications were reported, including cerebrospinal fluid fistulas, pneumonia, and even postoperative death due to pulmonary embolism [114]. This means that, although the method confirmed the technical feasibility of the procedure and the ability of the omentum to survive on the spinal cord, its unequivocal clinical efficacy has not been demonstrated.

The greater omentum has also been tested in selected brain pathologies. In experiments in dogs and other ischemia models, craniotomy was performed, and an elongated omental flap was transferred from the abdominal cavity to the surface of the brain and placed over the ischemic hemisphere [65,66,115]. In a canine model of cerebral ischemia, the presence of the omentum on the brain surface increased local blood flow and reduced the extent of infarction, which was explained by the formation of additional collateral circulation from the well-vascularized omental tissue [65,66]. These findings suggested that the beneficial effect of the omentum resulted primarily from improved perfusion rather than from direct neuronal regeneration. In humans, individual cases and small-group studies subsequently described the use of cerebral omentalization after ischemic stroke; however, these data remained limited and did not lead to the widespread adoption of this method in clinical neurology [116].

Similar assumptions were later explored in Alzheimer’s disease. In this approach, an elongated omental flap was surgically transferred to the surface of the brain, usually to the parietotemporal cortex, based on the assumption that increased blood flow and delivery of biologically active factors might support the function of damaged neurons [66,116,117]. Reports by Goldsmith (1996, 2002) [117,118] included individual cases and studies in small groups of patients, in whom transient or partial improvement in cognitive function after surgery was described [117,118,119]. However, these observations lacked control groups, carried a high risk of interpretative bias, and the method was not confirmed in comparative studies or established as a durable therapeutic option for neurodegenerative diseases. In practice, cerebral omentalization in Alzheimer’s disease has remained an experimental concept based more on biological rationale and selected clinical observations than on robust evidence of efficacy.

From a veterinary perspective, the importance of these reports lies primarily in demonstrating the biological potential of the greater omentum to support perfusion and healing within the central nervous system. At the same time, there are no data allowing this method to be considered clinically established in either humans or animals. In veterinary medicine, neurosurgical applications of the greater omentum therefore currently remain almost exclusively within the domain of experimental studies and translational considerations, rather than routine clinical practice.

### 2.6. Other Atypical Applications

Beyond the areas discussed above, the greater omentum has found a number of other less common applications. In the context of recurrent pericardial effusions, the concept of draining fluid from the pericardial sac into the peritoneal cavity has been developed using a pericardioperitoneal shunt [120]. This technique was palliative in nature and did not address the primary cause of the effusion, but it could reduce recurrences of tamponade by creating a permanent route for fluid drainage. In the clinical literature, mainly from human medicine, maintenance of shunt patency and good long-term control of recurrent effusions have been described, particularly in patients in whom conventional pericardiocentesis provided only temporary improvement [121,122]. From a practical perspective, this approach should be regarded more as a decompressive method than as causal treatment, while its current veterinary counterparts remain primarily pericardiocentesis, creation of a pericardial window, or subtotal pericardiectomy [121,123].

The omentum has also been used to wrap various vascular grafts and other implants in order to improve their integration and reduce the risk of infection [124,125]. Experimental studies have already shown that surrounding a prosthesis with well-vascularized omental tissue may limit the spread of infection and improve local healing conditions [12,34]. Later clinical reports and reviews from vascular surgery indicate that omentoplasty is still used as part of salvage management for infected grafts, especially when it is necessary to preserve or reconstruct vascular continuity in a high-risk infectious environment [126,127,128]. In this application, the omentum does not replace surgical debridement, replacement of infected material, or targeted antimicrobial therapy, but it may serve as a valuable adjunct by delivering well-vascularized tissue to the bed of the infected implant [128].

Concepts involving the use of the greater omentum to bypass the blood–brain barrier have also emerged. For this purpose, after resection of recurrent glioma, an autologous omental flap was transferred into the tumor cavity and revascularized, based on the assumption that newly formed vessels originating from the omentum would bypass the conventional blood–brain barrier and facilitate penetration of drugs and immune cells into the surrounding neural tissue [129]. Initial clinical reports demonstrated the technical feasibility of this procedure, and in a phase I study, it was considered safe and practicable. However, at the current stage, there are no data that allow its therapeutic superiority over standard neuro-oncological management to be clearly assessed [129,130]. This means that the use of the omentum in this indication remains experimental.

Some authors even describe the greater omentum as a type of bioimplant with strong proangiogenic and immunomodulatory potential, which may be useful in various extraperitoneal reconstructions [131]. In the veterinary literature, isolated case-based uses of the omentum have also been reported in exceptional situations, for example to close a spontaneous biliocutaneous fistula in a dog [132]. Such reports do not allow broader conclusions regarding efficacy to be drawn, but they clearly show that the greater omentum may be regarded as biologically active salvage tissue in unusual situations in which standard reconstructive methods are insufficient.

## 3. Limitations and Future Perspectives

At the current stage, extra-abdominal omentalization in small animals should be regarded as a specialized method requiring individual patient selection and appropriate surgical experience. The limitations of this technique result primarily from patient anatomy, including the limited reach of the pedicled flap, as well as from the need to perform laparotomy to harvest and shape the flap, which may prolong surgical time and increase operative trauma [24,27]. In clinical practice, this means that in difficult cases, such as recurrent abscesses, non-healing wounds, or non-union fractures in toy-breed dogs, omentalization should be considered as one of the therapeutic options rather than as a routine substitute for standard techniques [35,52,59,71]. With the development of microsurgery and minimally invasive techniques, the use of the greater omentum may become more accessible and safer for patients in the future. However, better-designed clinical studies are still needed to standardize indications and assess the true efficacy of this method [25,31,133,134].

Indocyanine green fluorescence angiography (ICG-FA) could be used intraoperatively to assess the perfusion and viability of the omental flap. Following intravenous administration of indocyanine green, near-infrared imaging enables real-time visualization of blood flow within the flap. In extra-abdominal omentalization, this assessment may be particularly useful after flap elongation and transposition, when vascular perfusion may be compromised by excessive tension, twisting, compression of the pedicle, or damage to small omental vessels. ICG-FA could help identify poorly perfused distal portions of the flap and guide intraoperative decisions regarding flap repositioning, release of tension, or removal of nonviable tissue before final fixation and wound closure. Although this technique is widely used for flap assessment in human reconstructive surgery [134,135,136], its application specifically to omental flaps in dogs and cats has not yet been validated and represents an area for future investigation. Future directions mainly involve combining the unique properties of the greater omentum with modern medical technologies. One such direction is the integration of the omentum with biomaterials and implants. Wrapping prostheses or scaffolds, for example vascular, esophageal, or tracheal implants, with an omental flap increases their vascularization, which may help reduce the number of complications, as demonstrated in experimental studies [137,138]. In bone tissue engineering, poor vascularization of grafts is considered one of the main limitations; therefore, concepts involving preliminary prevascularization using the omentum appear promising [139].

Another direction of development is tissue engineering and regenerative medicine. The adipose tissue stroma of the omentum contains mesenchymal stem cells, and the omental microenvironment supports the survival and activity of regenerative cells [12,13]. In an experimental canine model, the use of an omental flap together with periosteum and autologous adipose-derived stem cells (ADSCs) produced a synergistic effect and markedly improved reconstruction of a bone defect [57]. Further refinement of minimally invasive techniques may also be important. The development of minimally invasive surgery offers the possibility of reducing the trauma associated with harvesting and transferring the omentum. Laparoscopic harvesting of an omental flap has been described for coverage of an extensive soft tissue defect, significantly reducing the invasiveness of the procedure while maintaining reconstructive efficacy [133]. In the treatment of sternal infections in humans, an omental flap harvested and transferred using a minimally invasive thoracoscopic approach has also been used instead of conventional laparotomy [39]. Further improvement of endoscopic techniques may therefore reduce donor-site morbidity and make extra-abdominal omentoplasty more accessible.

## 4. Conclusions

The greater omentum is a unique anatomical structure with properties that are highly relevant to reconstructive surgery. It is richly vascularized and exhibits drainage, immunomodulatory, and proregenerative capabilities, with its effects associated, among other mechanisms, with enhanced angiogenesis and modulation of the inflammatory response at the site of injury. Although the role of the greater omentum is well established in human medicine, the potential for its use outside the peritoneal cavity in small animals has only recently begun to be analyzed more extensively. The available veterinary data are largely case-based or derived from small groups of clinical patients.

The literature review indicates that the greatest clinical value of omentalization may be expected in situations in which standard treatment is associated with a high risk of failure, for example in the presence of infection, extensive tissue defects and dead spaces, impaired vascularization, or a tendency for recurrence. In this context, its use has been described, among other sites, within the thoracic cavity, for example in chronic pleural and mediastinal abscesses, as well as in the treatment of infected chest wall wounds, where well-vascularized tissue may support infection control and improve local healing conditions.

However, the range of potential applications of the omentum extends beyond these areas. Data from human medicine, as well as anatomical and experimental studies, indicate the potential use of the omentum in head and neck reconstruction, for example in oncological surgery or in the treatment of selected fistulas. In experimental studies, transfer of the omentum to the spinal cord or brain has been associated with improved vascularization of damaged structures and a potential effect on neural tissue regeneration, representing an interesting direction for future research.

At present, extra-abdominal omentalization in small animals is not a routine procedure and requires both careful patient selection and appropriate surgical experience. Its limitations result primarily from patient anatomy and the need to perform laparotomy. In the future, the development of microsurgery and minimally invasive techniques may increase the accessibility of this method and improve its safety. However, better-designed clinical studies are still needed to standardize indications and reliably assess the efficacy of extra-abdominal omentalization in small animals.

## Figures and Tables

**Table 1:** Summary of the main extra-abdominal applications of the greater omentum in dogs and cats. The table presents typical clinical indications, biological and technical rationale, strength of available veterinary evidence, potential benefits, and the main limitations and risks associated with each application.

Area of Application	Typical Indications	Biological/Technical Rationale	Strength of Evidence (Veterinary)	Potential Benefits	Limitations and Risks
Thoracic cavity	Infections and tissue defects, dead space after resections, selected cases of chylothorax as an adjunctive procedure	Provides vascularized tissue, supports infection control and drainage, and fills dead space	Low to moderate, mainly case reports and case series [37,42,43,44,45,46,47,48,49,50,51]	Faster wound cleansing, improved filling of tissue defects, and potentially fewer recurrences in difficult cases	Prolonged surgical time; risk of abdominal complications; limited reach of the flap; the effect in chylothorax may be inconsistent
Orthopedics	Infected or infection-prone surgical fields, soft tissue defects around bones and joints, impaired or difficult healing	Angiogenesis, modulation of inflammation, and function as a “biological dressing”	Low, mainly case reports and experimental studies [52,53,54,55,56,57,58,59,60]	Support of tissue healing and vascularization, with potentially improved infection control	Lack of strong comparative studies; does not replace mechanical stabilization or control of the infectious focus
Head and neck	Tissue defects, difficult-to-heal wounds, dead spaces after resections	Fills defects and improves perfusion in compromised regions	Low [61,62,63]	Improvement of local healing conditions and protection of critical structures	Limitations related to flap reach and tunneling; risk of pedicle compression; technical difficulty
Neurosurgery/dural and peridural regions	Coverage of defects, support of tissue healing, reduction of dead space	Provides well-vascularized tissue in areas at high risk of infection and leakage	Low, sporadic reports [64,65,66]	Potentially improved healing and protection of neural structures	Difficult access and tunneling; lack of comparative data
Skin and wound reconstruction	Extensive wounds, chronic wounds, infected tissue defects	Acts as a “biological dressing”; supports angiogenesis and drainage	Low to moderate in terms of the number of available case reports and small case series; clinical outcomes are variable, and postoperative complications and wound recurrence are common [29,34,35,67,68,69,70,71,72,73]	Support of healing in compromised wound beds	Increased patient burden, longer surgical time, and local complications
Free grafting/microsurgery	Situations in which a pedicled flap cannot reach the target site	Allows placement in distant anatomical regions as free- non-vascularized graft or following microvascular reanastomosis	Very low in veterinary medicine; high technical barrier [25,28,52,54,58,60,70]	Possibility of use in distant recipient sites	Requires microsurgical expertise; risk of anastomotic thrombosis; prolonged surgical time

## Data Availability

No new data were created or analyzed in this study. Data sharing is not applicable to this article.
